# Primary Cutaneous Adenoid Cystic Carcinoma: A Misdiagnosed Tumor

**DOI:** 10.7759/cureus.64318

**Published:** 2024-07-11

**Authors:** Meryem Soughi, Sara Elloudi, Marco Ardigo, Hanane Baybay, FatimaZahra Mernissi

**Affiliations:** 1 Deparment of Dermatology, Centre Hospitalier Universitaire Hassan II, Fez, MAR; 2 Faculty of Medicine, Pharmacy, and Dental Medicine, Sidi Mohamed Ben Abdellah University, Fez, MAR; 3 Deparment of Dermatology, Istituto di Ricovero e Cura a Carattere Scientifico (IRCCS) Humanitas Research Hospital, Milan, ITA; 4 Department of Biomedical Sciences, Humanitas University, Milan, ITA

**Keywords:** adnexal tumor, immunohistochemistry, differential diagnosis, adnexal skin tumor, primary cutaneous adenoid cystic carcinoma

## Abstract

Primary cutaneous adenoid cystic carcinoma (PCACC) is a rare, slow-growing adnexal skin tumor with about 250 documented cases. We present a case involving a 66-year-old woman who was treated with ovulation inductors 30 years ago and underwent surgeries for meningioma 20 years ago and invasive galactophoric adenocarcinoma of the left breast 12 years ago. She presented with a gradually enlarging, solid, skin-colored tumor on her scalp, located along an old surgical scar initially diagnosed as a keloid by her surgeon. Clinical and dermoscopic evaluations suggested basal cell carcinoma or a metastatic tumor. Confocal microscopy showed deep infiltration without specific diagnostic clues. However, histopathological examination, immunohistochemistry, and comprehensive investigations confirmed the diagnosis of PCACC. A wide local excision was performed, with no recurrence noted during the two-year follow-up. This case highlights the challenges of diagnosing PCACC through clinical, dermoscopic, and confocal methods. Histological analysis remains essential, particularly to distinguish it from metastatic lesions, emphasizing the need for a thorough diagnostic approach in such cases.

## Introduction

Adenoid cystic carcinoma (ACC) is a rare form of cancer that originates in the salivary glands. ACC is called primary cutaneous ACC (PCACC) when it occurs exclusively on the skin, with no other primary site of malignancy in the body [1]. PCACC, a rare skin tumor first described by Boggio in 1975, has only 250 reported cases in the literature [2,3]. PCACC typically presents as slow-growing, painless, firm-to-cystic, skin-colored tumors measuring 0.5-8.0 cm in diameter. The scalp and chest are the preferred sites of location [4]. This tumor often occurs in middle-aged or elderly patients. There is a slight female predominance, with an average age of 59 years [5]. PCACC is characterized by a less aggressive evolution compared to the analog tumor in the salivary gland, and metastases are exceptionally common in PCACC [6]. We report a novel case of this rare tumor in a patient with a history of benign and malignant neoplasms, whose clinical and dermoscopic presentation posed significant diagnostic challenges.

## Case presentation

**Figure 1:**
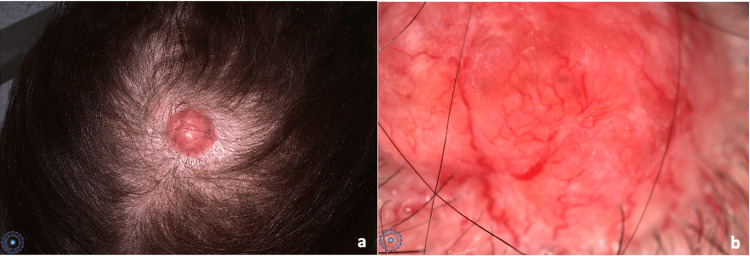
(a) A 2.5-cm erythematous nodule with a telangiectatic surface located on the vertex of the scalp. (b) Dermoscopy reveals a homogeneous erythematous background with arborizing vessels.

A 66-year-old woman, who was treated with ovulation inducers 40 years ago for infertility, underwent surgery for a meningioma 20 years ago and an invasive galactophore adenocarcinoma of the left breast 12 years ago. She is currently in remission. She presented to dermatology for skin cancer screening. Clinical examination revealed an erythematous nodule with a telangiectatic surface measuring 2.5 cm with alopecia, located on the vertex over the old surgical scar (Figure 1a), which had been evolving for five years and was initially treated as a keloid scar with topical corticosteroids. Dermoscopy showed a well-circumscribed homogeneous erythematous nodule, arborizing vessels with erosions, and hemorrhagic suffusions in some areas (Figure 1b).

**Figure 2:**
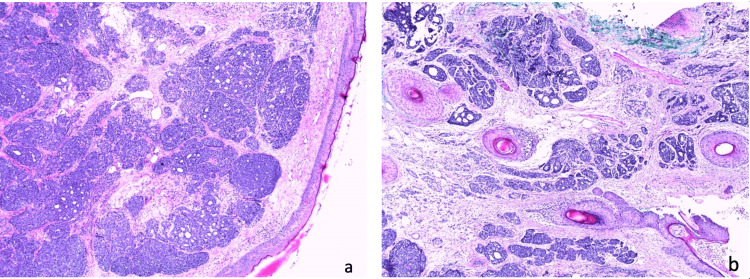
(a) Histological features at 50× magnification (H&E staining) showing a circumscribed neoplasm composed of clusters of basophilic cells with a cribriform pattern in the dermo-hypodermal region. (b) Histological features at 50× magnification (H&E staining) depicting cribriform and solid masses, as well as tubular structures lined by basaloid and myoepithelial cells.

**Figure 3:**
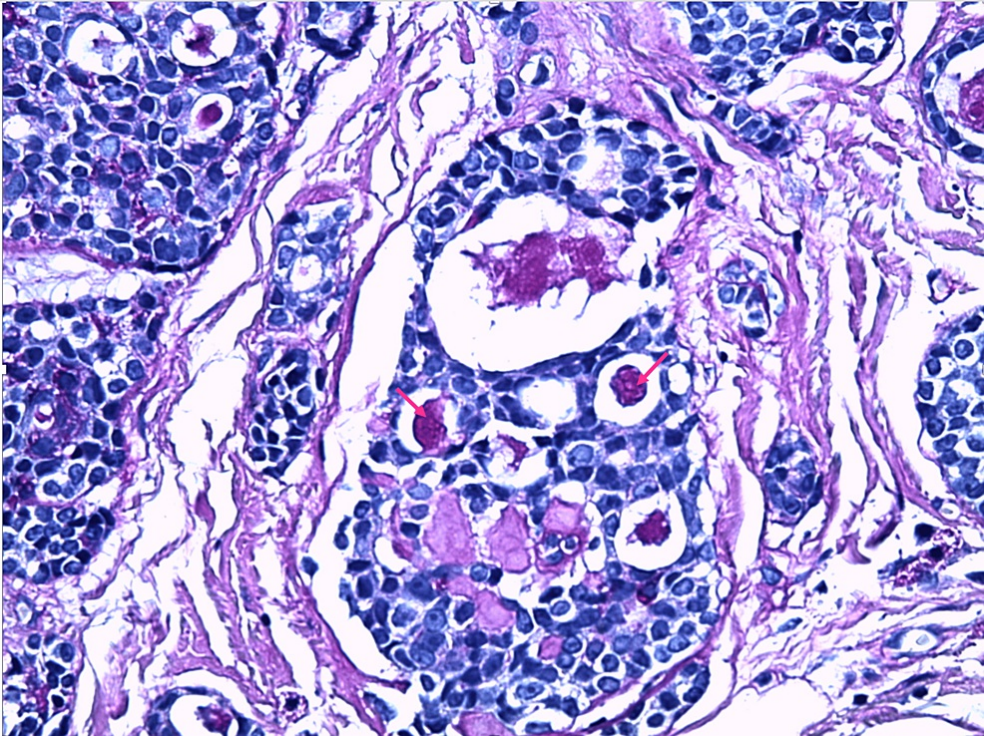
PAS staining at 400× magnification showing PAS-positive hyaline cylinders (pink arrow). PAS, periodic acid-Schiff

In reflectance confocal microscopy (RCM) images, glandular differentiation was not observed due to the deep proliferation, which is a limitation of RCM. However, a skin biopsy revealed neoplastic proliferation infiltrating the dermis and hypodermis. The tumor was characterized by cribriform and solid masses (Figure 2), along with occasional tubes containing periodic acid-Schiff-positive hyaline cylinders (Figure 3) and basophilic mucoid material. These tubes were lined with a double epithelial and myoepithelial layer. The tumor grew within a fibro-hyalinized, vascularized stroma, occasionally showing loose myxoid areas. Perineural tumor sheathing and infiltration of the arrector pili muscle were observed, with no vascular embolism detected.

**Figure 4:**
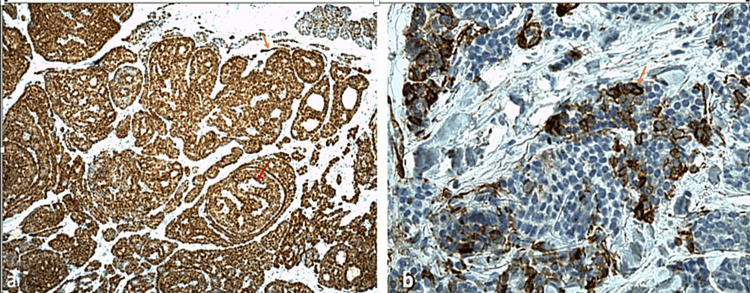
(a) Immunohistochemical staining at 100x magnification shows PS 100 expression in basaloid endoluminal cells forming cribriform masses with lumina (red arrow), as well as in myoepithelial cells lining the ducts (orange arrow). (b) Immunohistochemical staining at 400x magnification demonstrates focal expression of the smooth muscle actin antibody in myoepithelial cells (orange arrow).

**Figure 5:**
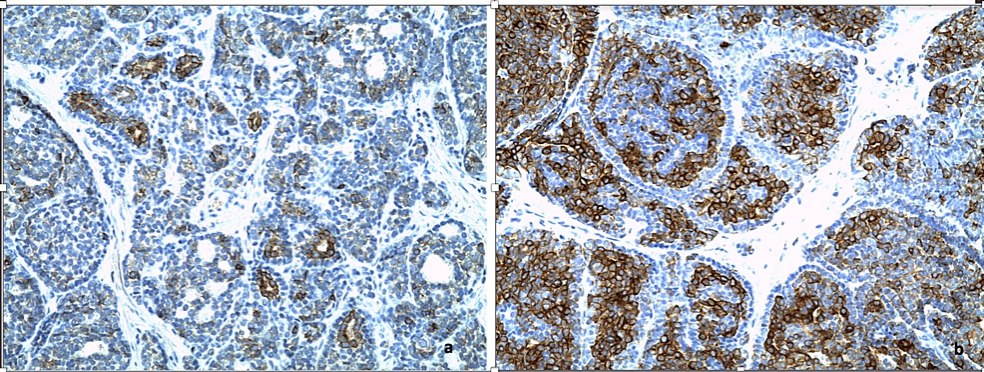
(a) Immunohistochemical staining at 200x magnification shows CD117 positivity (+) in endoluminal cells. (b) Immunohistochemical staining at 400x magnification reveals CK7 positivity (+) in endoluminal cells.

Immunohistochemistry (IHC) was conducted using the DAKO Auto Stainer Link 48 platform with the DAKO Envision K8000 detection system on deparaffinized sections. The results revealed the expression of anti-PS100 (Figure 4a), anti-CD117 (Figure 5a), anti-CK7 (Figure 5b), and anti-SOX10 antibodies in endoluminal and basaloid cells.

Myoepithelial cells were positive for anti-PS100 and anti-SOX10 antibodies and prominently for anti-smooth muscle actin antibodies (Figure 4b). Additionally, scattered cells expressed anti-P63, anti-p40, anti-DOG, and anti-GATA3 antibodies. Clinical examination and all radiological assessments, including PET-CT scans, showed no abnormalities. Based on the clinical, morphological, and immunophenotypic features described, the final diagnosis was PCACC. A wide excision was performed, with no recurrence observed during the one-year follow-up.

## Discussion

PCACC is an uncommon tumor, accounting for less than 1% of all head and neck cancers. [2]. The World Health Organization classifies it as an adnexal skin tumor [7]. The largest documented series, from the United States, included 152 cases of PCACC over 30 years, with an incidence rate of 0.23 per million person-years [8]. The precise etiology of ACC remains uncertain and is likely to arise from somatic mutations; however, the specific patterns of these mutations remain largely unexplored [9]. PCACC has been associated with other cancers, such as lymphohematopoietic and thyroid malignancies [8]. Conversely, some studies have found no association with hematopoietic cancers, suggesting that immunosuppression may not be a predisposing factor for PCACC [6,8]. In addition, autoimmune disorders such as Hashimoto’s thyroiditis, rheumatoid arthritis, and Sjögren’s syndrome have also been associated with PCACC [6]. In our patient, this cutaneous tumor appeared after years of cerebral and breast tumors, possibly linked to the hormonal treatments she received. This raises the question of whether this association is coincidental or if hormonal factors contributed to its development.

Additionally, our patient exhibited a typical presentation and location of this tumor - a slowly progressing scalp nodule. Initially suspected as cutaneous metastases from a primary tumor or basal cell carcinoma, dermoscopy revealed characteristic arborescent vessels typical of this tumor. However, RCM ruled out these diagnoses by revealing a deeper proliferation disconnected from the epidermis.

Certainly, the clinical diagnosis remains challenging due to its numerous differential diagnoses, such as the adenoid cystic variant of basal cell carcinoma, mucinous carcinoma, dermal cylindroma, and primary cutaneous cribriform apocrine carcinoma. Histologically, all these lesions closely resemble PCACC [5].

Histology remains the gold standard, revealing dermal tumor islands composed of basaloid cells arranged in a cribriform pattern within a fibrous, hyalinized stroma featuring pseudocysts containing mucin [4,10]. Notably, there is an absence of peritumoral clefting. PCACC typically lacks connection with the epidermis [5]. These tumors often exhibit cystic intratumor spaces containing alcian blue-positive mucin, a characteristic observed in our patient as well [11]. Perineural invasion, which was observed in our case, is present in approximately 76% of cases and is associated with an increased risk of local recurrence (46% vs. 22%) [11].

IHC reveals two distinct differentiation patterns: a prominent epithelial/ductal lineage characterized by positive staining for cytokeratin, S100 protein, and CEA; and a myoepithelial lineage marked by SMA, S100, and positive cytokeratin [5]. P63 expression is commonly observed in primary adnexal skin tumors but can also be sporadically found in visceral adenocarcinomas that metastasize to the skin [12]. CD117 expression, as observed in our case and reported in other studies, resembles that of mammary and salivary ACCs [6,13]. In our patient, the expression of CD117 and p63, as well as the mentioned markers, led to the need to use additional immunohistochemical markers such as anti-GATA3 for breast cancer metastases and anti-DOG1 for digestive cancer, taking into account the clinical context and the aforementioned observations.

PCACC is a locally invasive cancer. Among the 152 previously reported cases of PCACC, 70% of 130 PCACC cases had a localized stage, 25% had regional metastasis, and 5% had distant metastasis; the prognosis is better compared with ACCs of other organs that have a guarded prognosis [6,8].

Wide surgical excision with at least 2 cm tumor-free margins is advised to reduce the risk of local recurrence, in which perineural invasion plays a role after resection, and long‐term follow‐up for recurrence is recommended [3]. Mohs micrographic surgery has been used in a few cases [14]. Radiotherapy and chemotherapy can be used as adjuvant treatments, but no studies have demonstrated that radiation decreases the risk of local recurrence. Chemotherapy has been used in patients with distant metastases [4,14].

## Conclusions

PCACC represents a rare yet challenging entity in dermatological oncology, with a potential for misdiagnosis that underscores the importance of a multidisciplinary approach involving dermatologists, dermatopathologists, and oncologists. Accurate diagnosis through histopathological examination, IHC, and comprehensive investigations is crucial not only for diagnosis but also to rule out secondary localizations. While the prognosis is generally favorable with appropriate management, long-term follow-up remains essential due to the risk of late recurrences.
